# Inflammatory myofibroblastic tumor of the liver after adrenal neuroblastoma surgery: a case report

**DOI:** 10.1007/s12672-024-01039-4

**Published:** 2024-05-18

**Authors:** Qiyang Shen, Xingyu Liu, Lijie Zhang, Tao Li, Jianfeng Zhou

**Affiliations:** 1 Department of Pediatric Surgery, Children’s Hospital of Nanjing Medical University, Nanjing, Jiangsu China; 2 Department of Pediatric Surgery, First Affiliated Hospital of Bengbu Medical College, Bengbu, Anhui China; 3 Xuzhou Medical University, Xuzhou, Jiangsu China

**Keywords:** Neuroblastoma, Secondary tumor, Inflammatory myofibroblastic tumor, ICG, Whole transcriptome sequencing

## Abstract

A boy aged 55 months was diagnosed with stage IV Neuroblastoma (NB) of the right adrenal gland 2 years ago. Preoperative chemotherapy was given and he was then treated with retroperitoneal tumor resection and lymph node dissection. After surgery, the children were transferred to the Hemato-Oncology Department for chemotherapy according to the high-risk group NB, with outpatient follow-up every 6 months. In the second postoperative year, abdominal computed tomography (CT) scan revealed a rounded hypodense area in the upper part of the right posterior lobe of the liver, with marked inhomogeneous enhancement in the venous phase after enhancement, which was surgically resected, and postoperative pathology confirmed inflammatory myofibroblastic tumor (IMT) of liver. The patient was not given any special treatment after surgery. In this study, whole transcriptome sequencing was performed on the postoperative specimen of adrenal NB and the specimen of IMT of liver. This unusual case emphasizes the need for close monitoring of second tumor development in NB survivors even in the absence of known predisposing factors.

## Background

With improved diagnostic and treatment modalities for neuroblastoma (NB), the prognosis for patients is getting better, but the risk of patients developing a second tumor remains [1]. A so-called second tumor is another tumor that develops near or away from the primary tumor [2]. The appearance of a mass in the organism after the complete cure of a malignant tumor does not necessarily mean the recurrence of the tumor or the development of another malignant tumor [3]. In addition, the development of a second tumor may or may not be related to the treatment of the prior tumor, as genetic risk factors or other external carcinogens may also be involved [4]. The development of a second malignant tumor is one of the most devastating events affecting the survival of these patients [5, 6]. Studies suggest that the younger the cancer survivor, the more deadly the second primary malignancy [7]. Another study reported malignant gastrointestinal neuroectodermal tumour as the second primary malignant tumor after pediatric NB treatment [8]. IMT is a rare inert tumor that has been less frequently reported as a second tumor occurring after treatment of malignancy in pediatric patients, with cases of IMT occurring after treatment of pediatric nephroblastoma reported [9]. Characterized by spindle cell proliferation and inflammatory cell infiltration, IMT is an intermediate state tumor with malignant potential, and therefore should be given adequate attention when it appears as a second tumor [10]. This study reports a case of a child who developed liver inflammatory myofibroblastic tumor (IMT) after NB.

## Method

This study was reviewed by the ethics committee of the Children’s Hospital of Nanjing Medical University, batch number: NJCH2020137. The study was performed in accordance with the ethical standards as laid down in the 1964 Declaration of Helsinki and its later amendments or comparable ethical standards. The legal guardians/next of kin of the participants provided written informed consent for participation in this study. Written informed consent for the publication of any potentially identifiable images or data contained herein was obtained from the legal guardian/next of kin of the minor.

### Case presentation

**Fig. 1:**
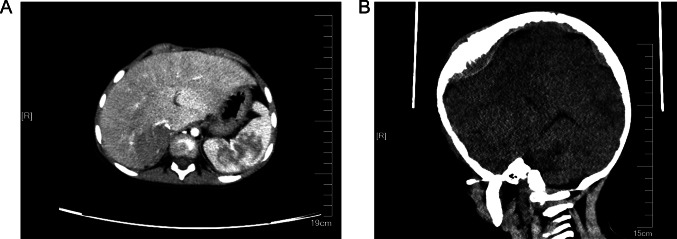
The patient’s first physical examination. **A** A whole-body CT shows a suspicious mass in the right adrenal gland. **B** CT scan of the head showed bony abnormalities of the frontal parietal bone with soft tissue masses on both sides, metastasis was considered

**Fig. 2:**
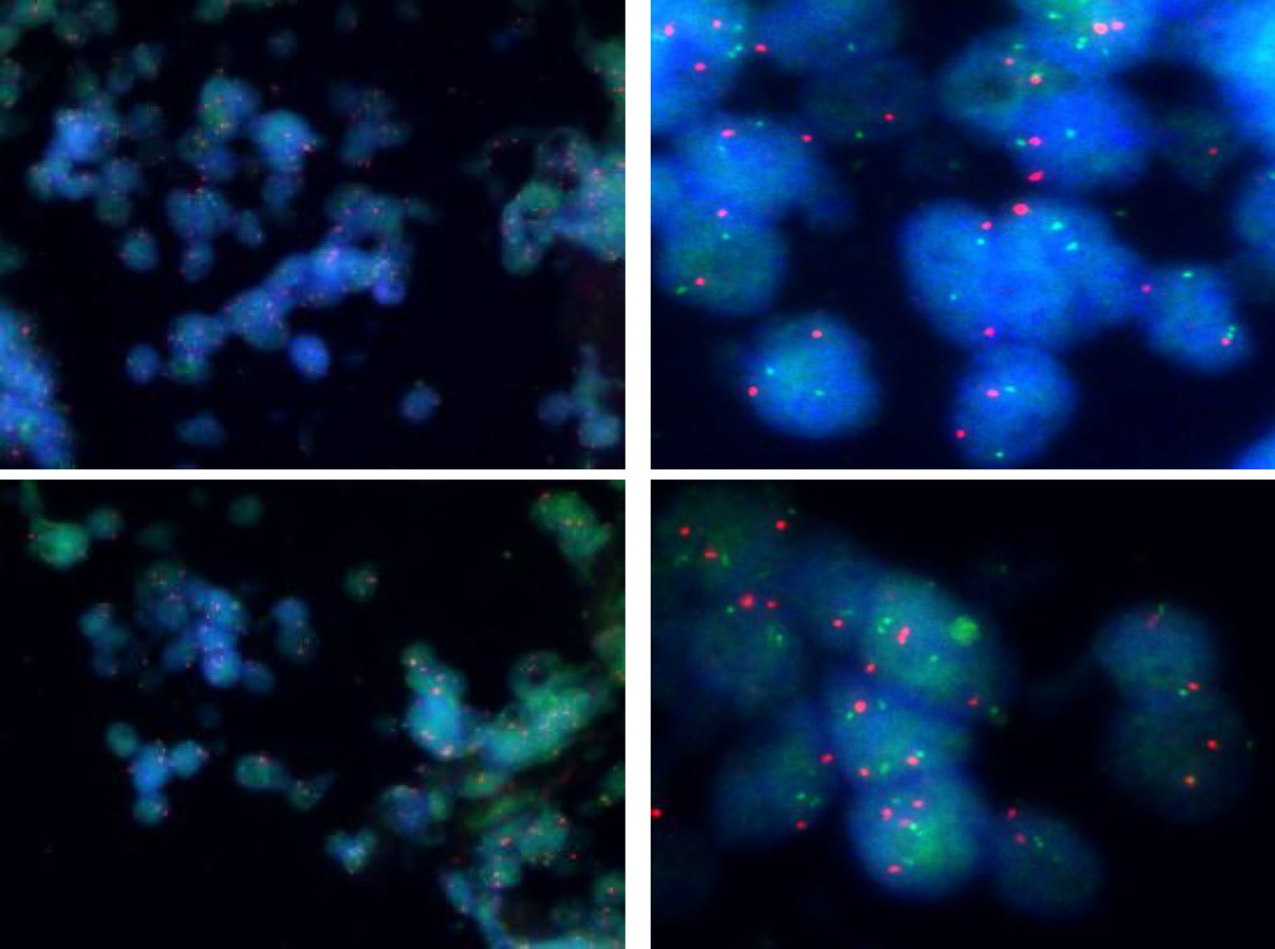
FISH assay to detect MYCN gene amplification, green signal for MYCN gene and orange gene for CEP2, × 1000. MYCN gene test result was gain. (Among the counted tumor cells, the MYCN/CEP2 ratio was 1.07, the mean MYCN signal was 2.45, and the mean CEP2 signal was 2.3)

A male child, aged 28 months, was admitted to the hospital for examination in 2018 when he was found to have a frontal parietal mass with fever for 6 days. Chest and whole abdomen CT scan showed soft tissue density mass shadow of the right adrenal gland, with multiple small nodular calcified foci seen internally; multiple nodular calcified foci seen in the retroperitoneum and pelvis, which was considered as NB with possible retroperitoneal pelvic multiple lymph node metastases (Fig. 1A). Cranial CT scan revealed thickening of the cranial plate of the frontal parietal bone on both sides, radiolucent periosteal reaction was seen in the inner plate, and soft tissue mass formation was seen in the surrounding area, metastasis was considered (Fig. 1B). The child underwent cranial tumor biopsy on 3/23/2018, and postoperative pathology showed a small round cell malignancy, and NB metastasis was considered in conjunction with the child’s clinical history and immunohistochemistry results. In this study, we used fluorescence in situ hybridization (FISH) to detect the amplification rate of the MYCN gene, and a MYCN (green signal)/CEP2 (orange signal) ratio > 4 was considered positive, i.e., MYCN amplification; a MYCN/CEP2 ratio ≥ 1 and < 4 was considered gain; and a MYCN/CEP2 ratio < 1 was considered negative. MYCN gene test result was gain (Fig. 2).

**Fig. 3:**
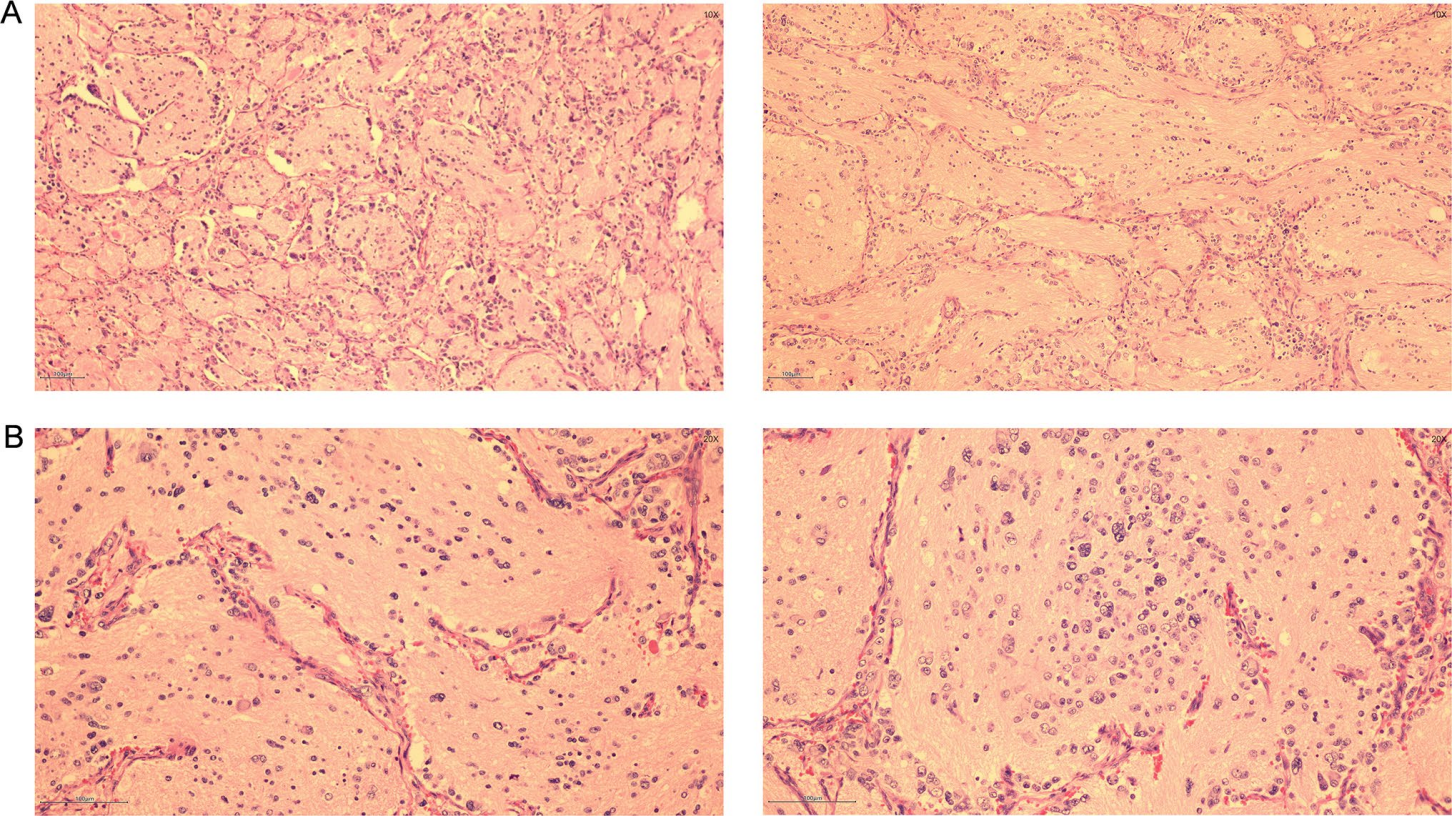
Hematoxylin and eosin stain of the mass. The patient had preoperative chemotherapy, and the postoperative NB pathological section results showed differentiated type (**A**: 40 × ; **B**: 100 ×)

The child was diagnosed with NB, stage IV, and was included in the ultra-high-risk group because of cranial, dural, and multiple vertebral metastases, and was subsequently transferred to the Hemato-Oncology Department for chemotherapy. Since 11/04/2018 patient was given 3 courses of chemotherapy in the ultra-high risk group regimen for NB (Course I: cyclophosphamide, topotecan; Course II: topotecan, cyclophosphamide; Course III: pedialyte glycosides, cisplatin). On June 29, 2018, the patient underwent retroperitoneal tumor resection and retroperitoneal lymph node dissection in the Department of General Surgery of Nanjing Children’s Hospital. Postoperative pathology revealed post-chemotherapy changes in NB (differentiated type) with lymph node metastasis (Fig. 3). Transferred to Hemato-Oncology for chemotherapy after surgery (Course 1: CTX + TOPO; Course 2: CTX + TOPO; Course 3: CDDP + VP16; Course 4: CTX + DOXO + VCR + MESNA; Course 5: CDDP + VP16; Course 6: CTX + DOXO + VCR + MESNA; Course 7: CTX + TOPO; Course 8: CDDP + VP16; Course 9: CTX + DOXO + VCR + MESNA; Course 10: CTX + TOPO) and the abdominal CT was reviewed regularly.

**Fig. 4:**
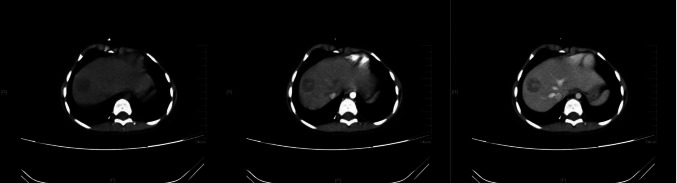
Abdominal CT showed that the liver was normal in shape and size, and the right lobe of the liver could be seen as a rounded hypodense area, with inhomogeneous enhancement seen in the venous phase, within which enhancement nodules could be seen

**Fig. 5:**
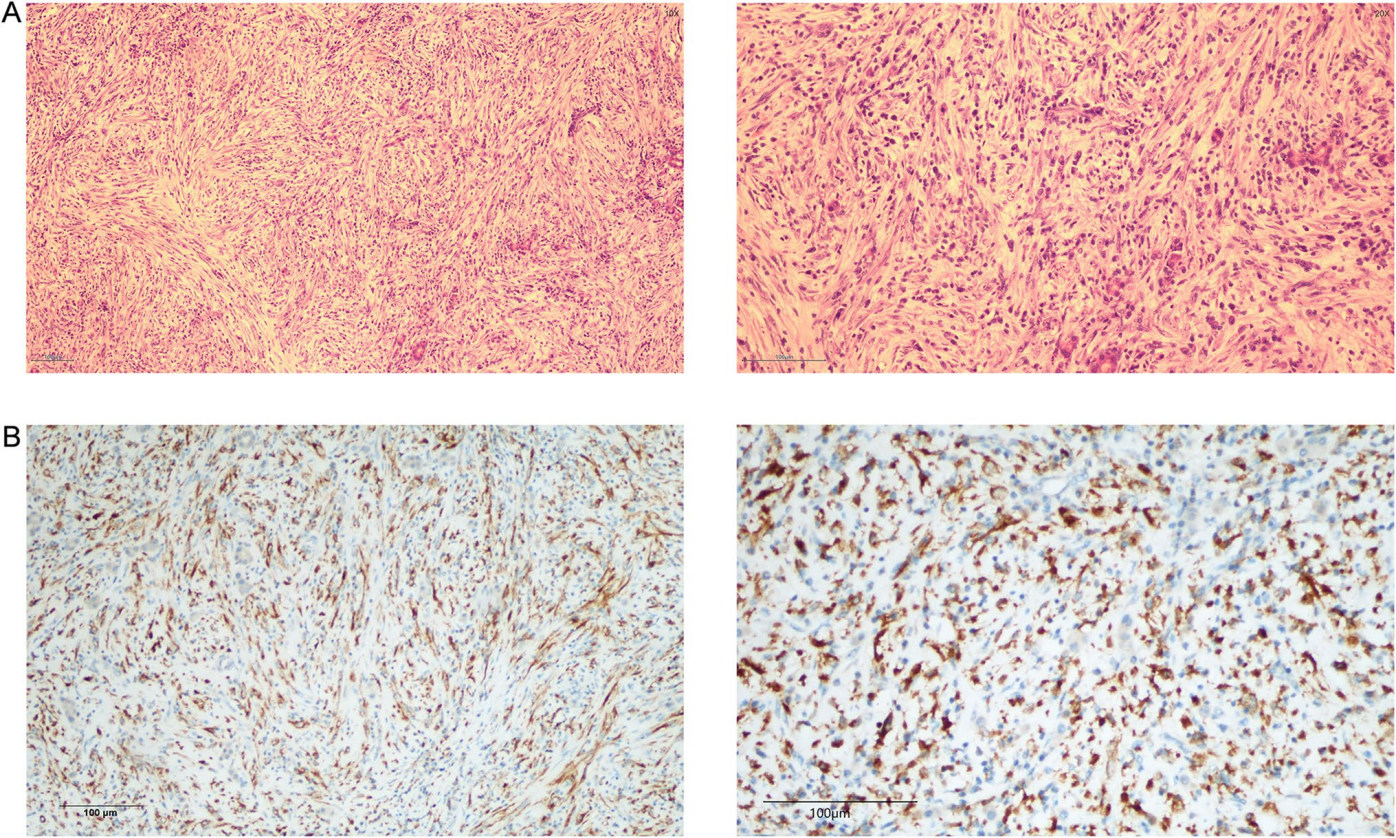
**A** Hematoxylin and eosin stain of the mass, postoperative pathology shows inflammatory myofibroblastic tumor (40 × ; 100 ×); **B** Immunohistochemistry for ALK shows strong positivity (100 × ; 200 ×)

**Fig. 6:**
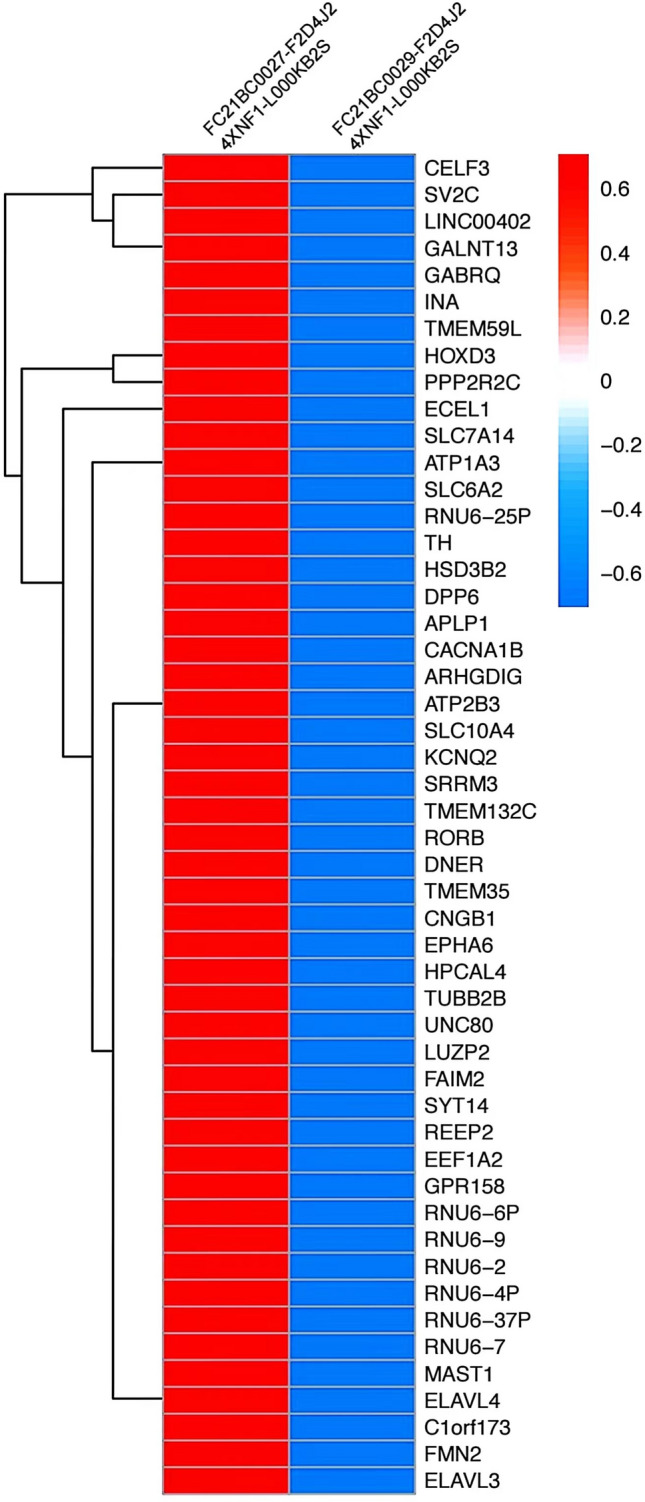
Whole transcriptome sequencing of adrenal NB specimens and liver IMT specimens revealed a large number of differential genes

The child underwent a routine postoperative thoracic and abdominal CT review in June 2020, which revealed an abnormal occupancy in the upper right posterior lobe of the liver. Further examination of the whole abdomen using CT scan with contrast showed that the right lobe of the liver was visible as a classically rounded low-density area with an extent of about 22 × 24 mm, and enhancement reveals inhomogeneous enhancement, which is evident in the venous phase, within which enhanced nodules are also seen.; NB liver metastasis is considered (Fig. 4). Subsequently, hepatic anomalous occupancy resection was performed in our General Surgery Department, and the occupancy was anatomically resected using indocyanine green fluorescence navigated hepatic occupancy resection, and postoperative pathology showed IMT (Fig. 5A). Ventana D5F3 immunohistochemical staining showed positive anaplastic lymphoma kinas (ALK), consistent with the diagnosis of IMT (Fig. 5B). Whole transcriptome sequencing of the adrenal NB specimen and the IMT of liver revealed a large number of differentially expressed genes (Fig. 6). A VIT chemotherapy regimen (Vincristine, Irinotecan and Temozolomide) was used postoperatively with outpatient follow-up.

## Discussion

Comprehensive treatment of NB is associated with multiple complications, including cognitive deficits, cardiotoxicity, and chronic kidney disease [1, 11, 12]. In addition, many individual cases have reported a second tumor after treatment [13, 14]. IMT of the Liver is a tumor-like lesion characterized by fibrous tissue, capillary hyperplasia, and local tissue inflammatory cell infiltration following necrosis of liver tissue caused by various inflammatory factors. The condition as a second tumor is reported for the first time [15].

When two tumors appear in adjacent locations at different times, multiple etiologies may be involved [16]. The second tumor may represent an extension or metastasis of the primary tumor [17]. Irradiation and drug therapy used as adjuvant modalities during the preoperative and postoperative period may have contributed to the development of the new tumor [18]. In addition, a patient’s genetic susceptibility to cancer, other environmental factors, or unknown causes may also lead to the development of a second tumor [19, 20]. Low-dose irradiation is known to induce a second tumor [21]. In general, the following criteria should be followed for the diagnosis of radiation tumors [22]: the new lesion appears in the radiated area; it is histologically distinct from the first tumor; it develops after a latent period that reasonably allows for the development of a secondary tumor, and there are no other conditions that predispose to tumor development. And the patient's medical records showed no other genetic or environmental factors predisposing him to cancer. Based on the fact that the patient received prior chemotherapy, the authors of this article do not exclude the possibility that chemotherapy may have acted as a causative factor in the development of hepatic IMT. However, it is not entirely clear whether this is the case.

In addition, in this study, whole transcriptome next-generation sequencing of adrenal NB specimens and IMT of liver specimens were performed. Whole transcriptome sequencing showed complete discordance in gene expression between adrenal NB specimens and liver IMT specimens, this transcriptomic level analysis again validated the non-homologous nature of the patient's two successive tumors. Genetic studies have similarly shown that rearrangement of the ALK gene may contribute to tumorigenesis, suggesting that IMT is likely to occur as a tumor entity and is not a reactive progression due to a particular disease [9]. Our immunohistochemistry results also showed strong positivity for ALK, which can be used to differentiate from NB. It is more likely that radiotherapy and medication used as adjuvant modalities during the preoperative and postoperative period contributed to the development of IMT of liver after adrenal NB surgery. Overall, the association between the two tumors awaits further study.

## Data Availability

The datasets for this article are not publicly available due to concerns regarding participant/patient anonymity. Requests to access the datasets should be directed to the corresponding author.
